# Entorhinal cortex-based metabolic profiling of chronic restraint stress mice model of depression

**DOI:** 10.18632/aging.102798

**Published:** 2020-02-12

**Authors:** Xi Chen, Tianlan Lan, Yue Wang, Yong He, Zhonghao Wu, Yu Tian, Yan Li, Mengge Bai, Wei Zhou, Hanping Zhang, Ke Cheng, Peng Xie

**Affiliations:** 1 Department of Neurology, Yongchuan Hospital of Chongqing Medical University, Chongqing 402460, China; 2 Department of Neurology, The First Affiliated Hospital of Chongqing Medical University, Chongqing 400016, China; 3 Chongqing Key Laboratory of Neurobiology, Chongqing 400016, China; 4 Institute of Neuroscience and the Collaborative Innovation Center for Brain Science, Chongqing Medical University, Chongqing 400016, China; 5 College of Biomedical Engineering, Chongqing Medical University, Chongqing 400016, China; 6 Institute of Neuroscience, Basic Medical College, Chongqing Medical University, Chongqing 400016, China

**Keywords:** depression, EC, metabolites, glutamate

## Abstract

Despite that millions of people suffer from major depressive disorder (MDD), the mechanism underlying MDD remains elusive. Recently, it has been reported that entorhinal cortex (EC) functions on the regulation of depressive-like phenotype relying on the stimulation of glutamatergic afferent from EC to hippocampus. Based on this, we used liquid chromatography-tandem mass spectrometry method to explore metabolic alterations in the EC of mice after exposed to chronic restraint stress (CRS). Molecular validation was conducted via the application of western blot and RT-qPCR. Through this study, we found significant upregulation of glutamate, ornithine aspartic acid, 5-hydroxytryptophan, L-tyrosine and norepinephrine in CRS group, accompanied with downregulation of homovanillic acid. Focusing on these altered metabolic pathways in EC, we found that gene levels of *GAD1*, *GLUL* and *SNAT1* were increased. Upregulation of SERT and EAAT2 in protein expression level were also validated, while no significant changes were found in TH, AADC, MAOA, VMAT2, GAD1, GLUL and SNAT1. Our findings firstly provide evidence about the alteration of metabolites and related molecules in the EC of mice model of depression, implying the potential mechanism in MDD pathology.

## INTRODUCTION

Major depressive disorder (MDD) is a severe disease which not only brings along physical and psychological pains in patients, but also results in heavy social and economic burden. Characterized with anhedonia and sustained mood despondency, MDD influences millions of people worldwide during their lifetime. Hypotheses about the potential etiology and pathogenesis of MDD have been put forward, like the disturbance of hypothalamic-pituitary-adrenal [1], loss of brain-derived neurotrophic factor [2] and decreased monoamine neurotransmitters [3]. Originated from early clinical observations, “monoamine hypothesis” of depression has played a crucial role in the development of antidepressant drugs [4]. Monoamine antidepressants, like selective serotonin reuptake inhibitors, serotonin and noradrenaline reuptake inhibitors and monoamine oxidase inhibitors, are widely used in clinical therapy of MDD patients [5]. However, the disparate effects of these drugs in clinical applications remain largely unknown. Still, the pervasive neurotransmitter disorder in MDD makes it crucial to explore the underlying mechanism. As reported in our previous researches, altered monoaminergic neurotransmitters were confirmed in depression models of mouse [6], rat [7] and non-human primate [8], providing direct evidence for monoaminergic disturbance in MDD. Aside from changes in monoamine transmitters (serotonin, dopamine and norepinephrine), glutamate, the main excitatory neurotransmitter in central nervous system, has been reported to be involved in the pathogenesis of depression [9]. We also validated that chronic social defeated stress influenced glutamate function in the prefrontal cortex [10]. Glutamate in the hippocampus of different rodent models of depression (chronic unpredictable mild stress, learned helplessness, chronic restraint stress (CRS) and chronic social defeat stress) was disturbed according to our previous research [11].

It is reported that hippocampus [12, 13] and its projected brain areas, like prefrontal cortex [14] and basolateral amygdala [15], are strongly related to MDD. However, it is recently identified that entorhinal cortex (EC), the main inputs of hippocampus, also plays a vital role in adjusting depressive-like behavior through the glutamatergic afferent connection with dentate gyrus [16]. Furthermore, volume decrease in EC was found in patients with remitted MDD [17]. Of note, although EC is traditionally reported to be correlated with Alzheimer's disease [18], it may function on the occurrence and development of MDD. Moreover, monoamine projections from midbrain and brainstem nuclei affect wide areas of cerebral cortices, including EC [19]. Hippocampus and EC are intimately interconnected via glutamatergic pathway [20]. Thus, it is viable and meaningful to explore whether there is metabolic alteration in the EC of mice with stress-induced depression and further to explore the potential molecular mechanism.

To confirm these hypotheses, we used liquid chromatography-tandem mass spectrometry (LC*-*MS/MS) method to detect the concentrations of 28 neurotransmitters in tryptophanic, GABAergic and catecholaminergic pathways. Key enzymes and transporters which function on the synthesis and decomposition of these neurotransmitters were validated through molecular approaches. In this study, we aimed to provide direct molecular evidence in the pathogenesis of MDD through comparing metabolic alterations in the EC of mice model of depression induced by CRS.

## RESULTS

### Evaluation of depressive-like phenotypes in CRS-treated mice

**Figure 1:**
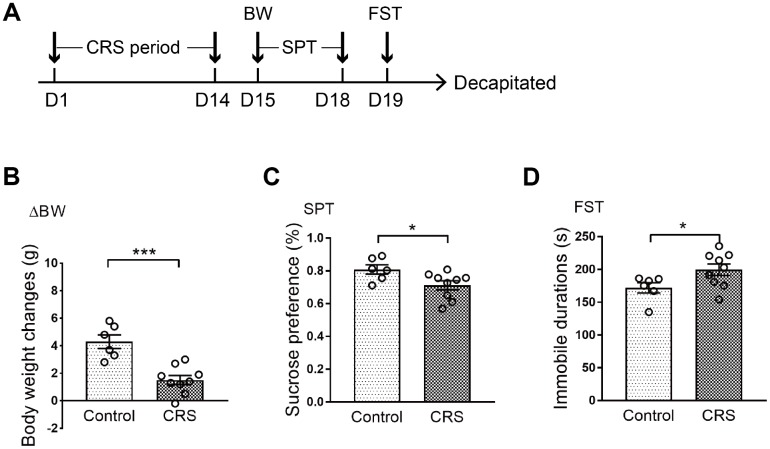
**Depressive-like behaviors in CRS mice.** (**A**) Schematic of the experimental process. The CRS process lasted 14 days. (**B**) The body weight changes of the two groups were displayed. (**C**) Sucrose preference was tested. (**D**) Immobile durations in FST after CRS. (* p<0.05, *** p<0.001; n=6-9 mice per group).

All mice were randomly divided into two groups with no significant difference in body weight. Processes of CRS and behavioral tests were conducted according to the schematic (Figure 1A). Body weight of each mouse was measured on the last day of CRS. It was shown that body weight changes in CRS group were significantly lower than control group after two weeks of experiment (P<0.001; Figure 1B). Then, depressive-like phenotypes were evaluated through sucrose preference test (SPT) and forced swim test (FST). Obviously, sucrose preference in CRS group was decreased when compared with control group (P=0.033; Figure 1C). Moreover, immobile durations in FST were obviously prolonged in CRS group (P=0.047; Figure 1D).

### Detection of neurotransmitters in the EC of CRS mice

**Figure 2:**
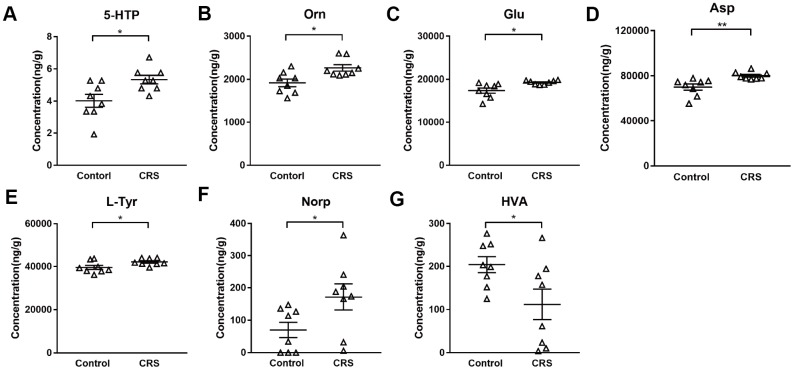
**Altered metabolites in the EC of CRS-treated mice.** Levels of metabolites (**A**) 5-HTP (**B**) Orn (**C**) Glu (**D**) Asp (**E**) L-Tyr (**F**) Norp (**G**) HAV were calculated in control and CRS groups with data in LC-MS/MS. (* P <0.05, ** P <0.01; n=8 mice per group).

Levels of 28 neurotransmitters were detected in EC tissues of both groups. As shown in Table 1, except for 7 neurotransmitters (tryptamine, 5-hydroxytryptamine, 3-hydroxyanthranilic acid, phenylethylamine, tyramine, dopamine, vanillylmandelic acid) which were too low to be detectable, levels of 21 neurotransmitters were measured in both groups. Compared with control group, only 5-hydroxytryptophan (5-HTP; P=0.016; Figure 2A) was upregulated in tryptophanic pathway. Still, ornithine (Orn; P=0.011; Figure 2B), glutamic acid (Glu; P=0.017; Figure 2C) and aspartic acid (Asp; P=0.008; Figure 2D) in CRS group were all upregulated in GABAergic pathway. Moreover, in catecholaminergic pathway, L-tyrosine (L-Tyr; P=0.036; Figure 2E) and norepinephrine (Norp; P=0.046; Figure 2F) were both upregulated in contrast to the significant downregulation of homovanillic acid (HVA; P=0.042; Figure 2G) in the EC of CRS mice.

**Table 1:** Concentration(ng/g) of neurotransmitters in EC.

**Pathway**	**Metabolite**	**Control group**	**CRS group**
**Tryptophanic pathway**	Trp	1878.48±143.38773	2001.24±83.09105
	5-HTP	4.02±0.40504	5.34±0.26349*
	NAS	6.66±1.04392	7.26±0.77205
	Trpo	3.06±0.28596	2.58±0.33865
	5-HIAA	51.24±3.98072	51.96±6.48619
	KYNA	0.78±0.08783	0.72±0.09071
	Ind-3-C	1.20±0.18142	1.26±0.18
**GABAergic pathway**	Orn	1920.24±89.27433	2263.32±74.41923*
	Gln	13760.76±738.15	12750.90±167.77
	GABA	7121.58±262.95505	7558.38±190.488
	a-KG	15.66±2.88134	19.08±2.50033
	Glu	17408.04±611.15432	19302.30±162.78369*
	GSH	15743.10±1820.16229	15045.30±355.08446
	SA	1.44±0.70848	6.66±2.30099
	Asp	70090.86±2735.29988	80122.32±1138.30867**
**Catecholaminergic pathway**	L-Phe	3149.70±51.54419	3005.22±176.54411
	L-Tyr	39582.18±944.78753	42143.04±567.50002*
	L-DOPA	1917.66±362.02586	1931.82±45.42842
	DOPAC	502.32±47.07331	473.16±29.71085
	Norp	69.84±23.74701	171.90±40.10108*
	HAV	204.06±18.58311	111.00±35.25847*

*P<0.05 **P<0.01.

Abbreviations: Trp: tryptophan; 5-HTP: 5-hydroxytryptophan; NAS: N-acetyl-serotonin; Tra: tryptamine; Trpo: tryptophol; 5-HT: 5-hydroxytryptamine; 5-HIAA: 5-hydroxyindoleacetic acid; KYNA: kynurenic acid; 3-HA: 3-hydroxyanthranilic acid; Ind-3-C : indole-3-carboxaldehyde; Orn: ornithine; Gln: glutamine; GABA: γ-aminobutyric acid; α-KG: α-ketoglutaric acid; Glu: glutamic acid; GSH: glutathione; SA: succinic acid; Asp: aspartic acid; L-Phe: L-phenylalanine; PEA: phenylethylamine; L-Tyr: L-tyrosine; Trya: tyramine; L-DOPA: L-3,4-dihydroxyphenylalanine; DOPN: dopamine; DOPAC: 3,4-dihydroxyphenylacetic acid; VMA: vanillylmandelic acid; Norp: norepinephrine; HAV: homovanillic acid.

### Altered gene expression in EC

**Figure 3:**
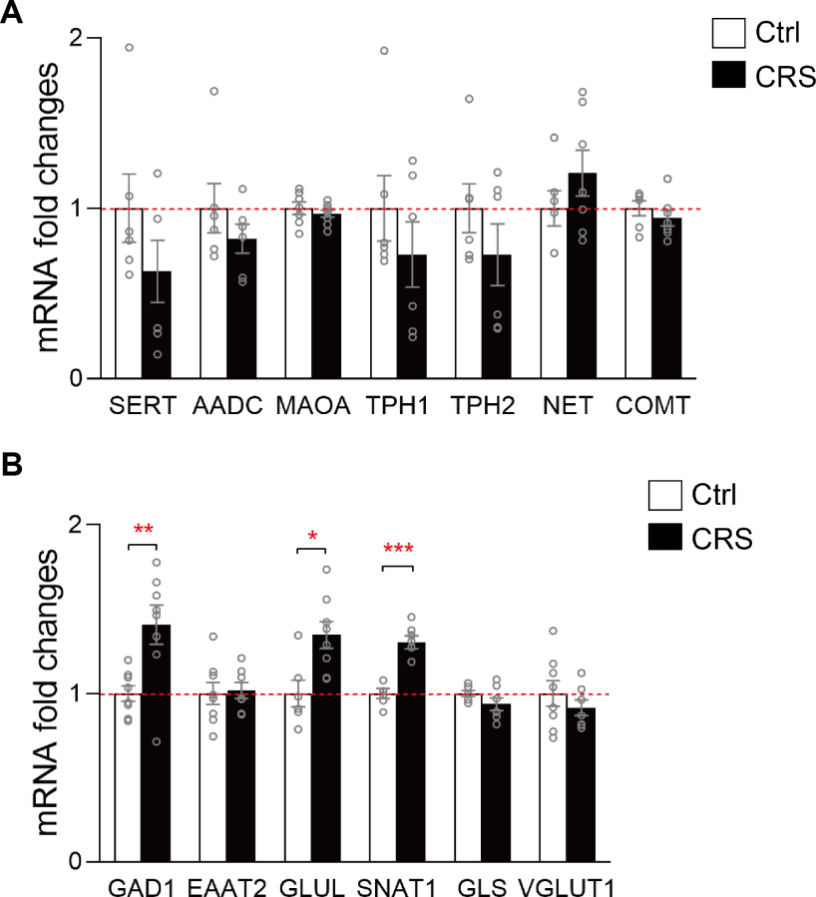
**RT-qPCR validation of mRNA expression levels of key enzymes and transporters in glutamatergic and monoaminergic pathways in the EC of mice after CRS exposure.** β-actin and GAPDH were used to normalize the expression levels of genes in control group and CRS group. (**A**) The mRNA expression levels of *SERT*, *AADC*, *MAOA*, *TPH1*, *TPH2*, *NET* and *COMT* in monoaminergic pathway were examined by RT-qPCR. (**B**) The mRNA expression levels of *GAD1*, *EAAT2*, *GLUL*, *SNAT1*, *GLS* and *VGLUT1* in glutamatergic pathway were tested in both groups through RT-qPCR. (* P<0.05, ** P<0.01, *** P<0.001; n=6-8 mice per group).

Based on previous results and KEGG analysis, we then further verified genes related to the synthesis and metabolism of altered neurotransmitters in monoaminergic and glutamatergic pathways in EC. The mRNA levels of key enzymes and transporters in the two pathways were validated via RT-qPCR. No significant differences were found in mRNA expression levels of *SERT*, *AADC*, *MAOA*, *TPH1*, *TPH2*, *NET* and *COMT* in monoaminergic pathway (Figure 3A). However, in glutamatergic pathway, we found that *GLUL*, which regulated the transformation of glutamate into glutamine in astrocyte, was elevated in the EC of CRS mice (P=0.01; Figure 3B). The transcription of *GAD1* in CRS group showed a significantly upregulation compared to control group (P=0.006, Figure 3B). Also, mRNA level of *SNAT1* was increased in depressed mice (P<0.001, Figure 3B). Still, we found no significant changes of mRNA expression levels in *EAAT2*, *GLS*, *VGLUT1* (Figure 3B).

### Altered protein expression in EC

**Figure 4:**
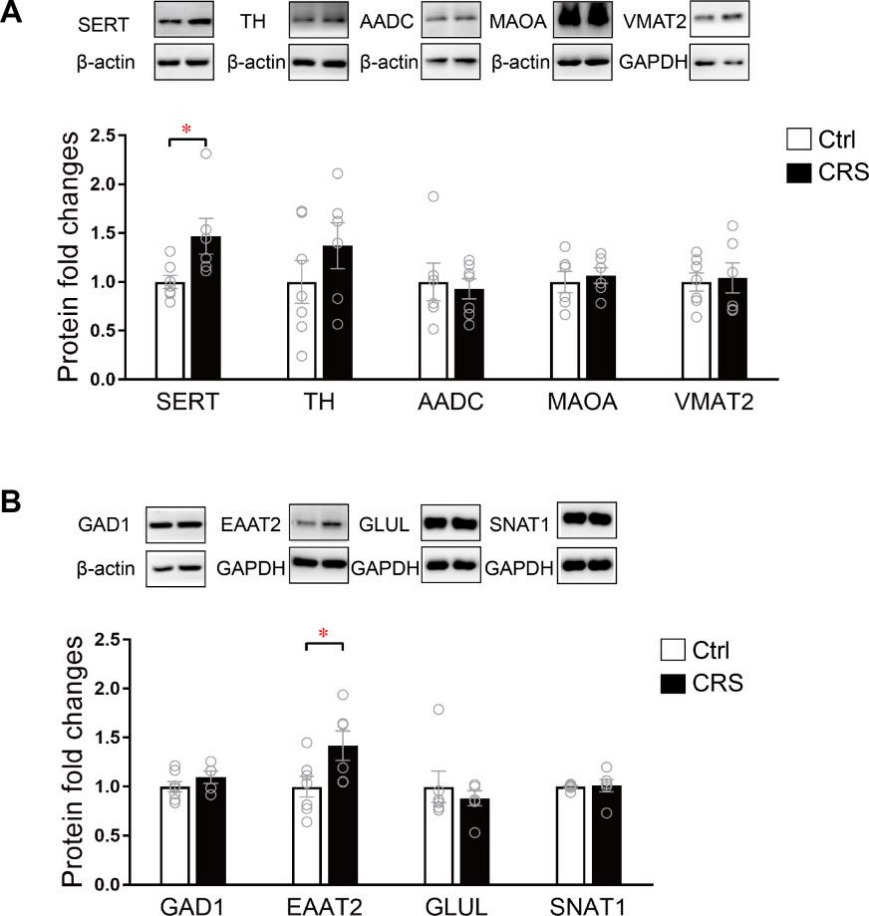
**Western blot analysis of protein expression levels of key enzymes and transporters in glutamatergic and monoaminergic pathways in the EC of mice after CRS exposure.** β-actin and GAPDH were used to normalize the expression levels of proteins in control group and CRS group. (**A**) The protein expression levels of SERT, TH, AADC, MAOA and VMAT2 in monoaminergic pathway were examined by western blott (* P<0.05, n=6-7 mice per group). (**B**) The protein expression levels of GAD1, EAATT2, GLUL and SNAT1 in glutamatergic pathway were analyzed in both groups. (* P<0.05; n=6-7 mice per group).

The protein expression levels of SERT, TH, AADC, MAOA, VMAT2, GAD1, EAAT2, GLUL and SNAT1 were further validated by western blot. As presented, protein expression of SERT in monoaminergic pathway was significantly increased in the EC tissue of CRS mice (P= 0.02; Figure 4A). Protein expression of EAAT2 in glutamatergic pathway was found upregulated in CRS group (P=0.04; Figure 4B), whereas no changes were found in protein expression levels of TH, AADC, MAOA, VMAT2, GAD1, GLUL and SNAT1 (Figure 4).

## DISCUSSION

**Figure 5:**
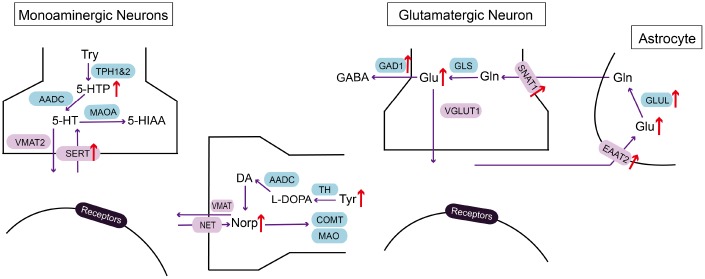
**Summarizing schematic of identified molecular alterations in EC after CRS process.** Alterations in metabolites and related molecules in monoaminergic and glutamatergic pathways in the EC of CRS mice (red arrow) were presented. Targeted metabolomics was used to measure altered metabolites. Molecular approaches were used to verify changes of key enzymes and transporters in monoaminergic and glutamatergic pathways.

With the application of LC-MS/MS in investigating neurotransmitter alteration in the EC of CRS-induced depressed mice, we demonstrated that 5-HTP, Orn, Glu, Asp, L-Tyr, Norp were significantly elevated compared with those of control group, while HVA was reduced (Figure 5). These results indicated that the disorder of metabolites may be involved in the process of MDD in EC.

Several lines of evidences have revealed glutamate elevation in plasma, cerebrospinal fluid and cortex of postmortem human brains [21, 22]. The stimulation of glutamatergic afferent in EC has been proved to be antidepressant through dentate gyrus activity-dependent process [16]. Therefore, we wonder the underlying molecular mechanism of glutamate disorder in the EC of mice after CRS process. We verified the alteration of key enzymes and transporters in glutamatergic pathway with RT-PCR and western blot. In addition, we simultaneously investigated molecules in monoaminergic pathway because of the upregulation of 5-HTP and Norp. As a result, *GAD1*, *SNAT1* and *GLUL* in the EC of CRS group were all increased at transcriptional level together with the elevation of protein expression in SERT and EAAT2, suggesting molecular mechanisms of glutamate and monoamine disturbance in the EC of depressed mice.

GAD1 is the key enzyme participating in the synthesis of GABA with glutamate. Our metabolomics data showed that glutamate elevated in depression group, while GABA didn’t. This disequilibrium may be a potent cause of exitotoxicity in the brain [21]. Hence, we verified the upregulation of *GAD1* in mRNA expression level in the EC of mice treated with CRS, which may be an outcome of compensation mechanism to avoid the neurotoxicity of glutamate. The unchanged expression level of GAD1 protein may be correlated to various reasons, like limited local available resources for protein biosynthesis, enhanced process of protein decomposition and response delay in translation [23]. Previous research showed that the neuropil relative density of GAD1 was increased in the EC of MDD patients under the application of in situ hybridization [24]. On the contrary, no *GAD1* abnormalities in mRNA level was found in the EC of MDD patients [25]. The contradictory results in reported research and our findings may be due to the different sensitivity of experimental methods adopted. SNAT1 supplies glutamatergic neurons with glutamine which is required for the synthesis of glutamate [26]. GLUL catalyzes the ATP-dependent conversion of glutamate to glutamine. At protein level, we found that EAAT2 was significantly increased compared with control group, which may induce the clearance of raised extracellular glutamate in the brain. Therefore, the increased *SNAT1* and *GLUL* mRNA and EAAT2 protein in our findings may facilitate the cycle between glutamate and glutamine, resulting in glutamate accumulation in EC.

In monoaminergic pathway, we confirmed that protein expression of serotonin transporter, SERT, was specifically increased in the EC of CRS group, even though the mRNA level of *SERT* remained unchanged, which may be related to the enhanced translational efficiency and decreased process of protein degradation. SERT ensures the reuptake of serotonin in synaptic cleft. As proposed in monoamine hypothesis, low level of serotonin in MDD patients is the target of antidepressant drugs, like SSRIs, which exert antidepressant effect through reducing SERT expression in serotonergic raphe nuclei via binding to SERT [27]. Therefore, the elevation of SERT in the EC of CRS group is helpful in promoting the clearance of serotonin in synaptic cleft, which may be the early phase reaction before low serotonin.

Even though we provide direct evidence of metabolite disorders in the EC of depressed mice, there are also limitations in our present study. The underlying reasons of inconsistent molecular changes in protein and mRNA are not demonstrated experimentally. It remains to find out whether these significant molecular changes are vital downstream effectors and even can be reversed by antidepressant drugs. Also, it remains unknown whether the upregulation and downregulation of molecules found in our research can induce depressive-like behaviors. Further researches are needed to explore complicated mechanisms of EC in MDD. Despite these limitations, our findings firstly shed light on the participation of EC in neurotransmitter disorder of depression, which may be a pharmacological potential for MDD.

Related to previous study [16], we mainly focused on glutamate disturbance in our findings. Although it has been proposed that stress-induced depressive-like behaviors can be ameliorated by chronic stimulation of glutamatergic neurons in EC, it has thus been unclear whether glutamatergic neurons are excited in EC after stress-induced process. Interestingly, we show that stress-induced upregulation of glutamate may lead to the stimulation of neurons in EC, which may be a compensating mechanism to against the chronic stress. Chronic stress induced glutamate upregulation was also found in hippocampus, as proposed in previous study [11]. Furthermore, this compensatory production of glutamate may spur the circuit of EC-DG to drive antidepressant behavior. Therefore, our results indicate that the enhancement of excitatory neurons through the secretion of glutamate in EC may be a protective mechanism in face of stressful situations.

Collectively, our study validated the first evidence about neurotransmitter disturbance in the EC of mice with CRS-induced depression. Molecular alterations in glutamatergic pathway, like GAD1, EAAT2, SNAT1 and GLUL, may aid to explain the antidepressant effect of glutamatergic stimulation in EC [16]. With the accumulation of glutamate in EC-DG circuit, targeting this circuit may provide a new treatment for MDD and will be a promising research field in exploring mechanism of MDD.

## MATERIALS AND METHODS

### Animals

Male adult C57BL/6 mice (8-16 weeks of age; weighing around 20g) were used for establishing CRS model of depression. Animals were housed under relative steady conditions (12h light-dark cycle at a humidity of 55± 5% and constant temperature of 23± 2°C) with adequate water and food. All animals were purchased from the experimental animal center of Chongqing Medical University (Chongqing, China). All animal experimental procedures of this study were approved by the Ethics Committee of the Chongqing Medical University (Approval No. 20160331). Care and treatment of animals were in accordance with the requirements of the National Institutes of Health Guidelines for Animal Research.

### Chronic restraint stress

Animals were exposed to CRS by placing in 50ml-plastic tubes with a few holes to keep air flow for 4-8h per day. The total process continued 14 consecutive days beginning at 11:00am each day, while the control group received the same time for food and water depriving without restraint. Body weight of each mouse was measured on the last day of chronic restraint.

### Behavioral assays

Behavioral analysis was performed by testers blinded to experimental conditions. Experiments were done during the light phase of mice in a soundproof room. All the animals' behaviors were videotaped and measured by video tracking software (Smart).

### Sucrose preference test

The sucrose preference test was described in our previous research [28]. Mice were single housed and habituated to 1% sucrose solution and water for 2 days without any stress. Bottle positions were switched at the 24^th^ hour. After that, mice were food and water deprived for 12h. Mice were exposed simultaneously to two bottles for 12h in the dark phase, one containing with 1% sucrose solution and the other with tap water. Total consumption of each fluid was measured and sucrose preference ratio was calculated through dividing the total consumption of sucrose by the total consumption of both water and sucrose.

### Forced swim test

Mice were placed separately in a transparent glass cylinder filled with 20cm tap water (23 ± 1°C). The first 2 minutes was used for adaptation and the duration of immobility was measured automatically in the next 4 minutes by video-tracking system. Tap water in cylinder was changed after each trial.

### Sample collection and pretreatment

After all behavioral tests completed, all mice were anesthetized with 1% pentobarbital sodium and EC tissues were separated quickly with a brain matrix. Samples were frozen in liquid nitrogen and stored at -80 °C before biochemical analysis.

### LC-MS/MS

The process of LC*-*MS/MS analysis was conducted as previously described [6]. In short, EC tissue samples were diluted in 400 μL methanol-water mixed solution (4/1, v/v, 0.1% formic acid) containing 75ng/ml L-Phe (2Cl)-OH as an internal standard. About 300ul upper solution was collected after centrifugation (14000 g, 10 min, 4°C). Then the residue solution was mixed with 200μl the same methanol-water mixed solution (1/1, v/v, 0.1% formic acid) and upper solution was collected after centrifugation (14000 g, 10 min, 4°C). The extractions were then concentrated and resolved in methanol-water mixed solution (1/1, v/v, 0.1% formic acid) for LC-MS analysis. We used Waters Xevo® TQ-S mass spectrometry (MS) system with Waters ultra-performance liquid chromatography (UPLC) amino column (2.1 mm × 100 mm, 1.7μm) to separate the metabolites (50°C, 1μL sample injection). The data were finally analyzed by MassLynx4.1 software.

### RT-qPCR

Total RNA of EC tissue was extracted via the application of Trizol reagent (Invitrogen). cDNAs were synthesized using PrimeScript™ RT reagent Kit (TAKARA). The RT-PCR reactions were conducted with a SYBR green detection system (Promega). We used β-actin and GAPDH as housekeeping genes to normalize the data. All primers were exhibited in Table 2. 2-ΔΔCT or Pfaffi methods were applied to analyze these results [29].

**Table 2:** Primer sequences used in RT-qPCR.

**Gene**	**Forward Primer (From 5′to 3′)**	**Reverse Primer (From 5′to 3′)**
GAD1	TCACCTCAGAACACAGTCACT	TTCCCCCTTTCATTGCACTTT
GLS	TTATGCCACTGTTTCTGCTG	GGTTATCAAGTCCCTGACGG
EAAT2	TGCCAACGGAGGATATCAGTCT	CTGCATTCGGTGTTGGGAGTC
VGLUT1	CGTGTCCATGGTCAACAACAG	ACAGTCTCTGGATCCCAGTTGAA
SNAT1	AACCCGGCCTTTTACCTTCC	CCCGGCAGTTAGATGTCCTT
GLUL	TGGTCTGAAGTGCATTGAGGAG	CGGCAGAAAAGTCGTTGATGTT
AADC	CAGTCCTCCTCTTCACCC	CCACATCCTGCTGTTCTT
TPH1	GGGCTTGACTTTGTCTCTGC	GTTTGAATCTGGCCTGGTGT
TPH2	CCTACACGCAGAGCATTGAA	CTAGGCATCAAATCCCCAGA
MAOA	GTATGGAAGGGTGATTCGGCA	ACTGCACCTTCCATGTAGCC
SERT	GCTGAGCTGACTTGGATA	ACAGACGTTCACAGACCTAA
NET	TGCACGAGAGCAGTGGGAT	CGACCATCAGGCAGAGCAG
COMT	ACCGCTACCTTCCAGACA	GCCGTCCACCACTTTCAT
β-actin	GCCACCAGTTCGCCATGGAT	TCTGGGCCTCGTCACCCACATA
GAPDH	ACCCAGAAGACTGTGGATGG	CACATTGGGGGTAGGAACAC

Abbreviations: GAD1: glutamate decarboxylase 1; GLS: glutaminase; EAAT2: excitatory amino acid transporter 2; VGLUT1: vesicular glutamate transporter 1; SNAT1: sodium-coupled neutral amino acid transporter 1; GLUL: glutamine synthetase; AADC: aromatic-L-amino-acid decarboxylase; TPH1/2: tryptophan 5-hydroxylase ½; MAOA: monoamine oxidase A; SERT: sodium-dependent serotonin transporter; NET: norepinephrine transporter; COMT: catechol O-methyltransferase; GAPDH: glyceraldehyde-3-phosphate dehydrogenase.

### Western blot

The protein of EC was extracted as previously described [10]. Tissues were homogenized in RIPA buffer (Beyotime) containing protease inhibitor cocktail tablets (Roche). After the measurement of the concentration of protein by BCA assay, 10–20μg proteins of each lane were separated on a 10% SDS–PAGE gel and then transferred to polyvinylidene fluoride membrane (Millipore). After blocking in 5% no-fat milk powder for 2h at room temperature, the membranes were then incubated with primary antibodies (Table 3) overnight at 4°C. All primary antibodies used were diluted in primary antibody dilution buffer (Beyotime). Then, these membranes were washed with Tris-buffered saline 0.05% Tween-20 (TBST) for three times and reacted with secondary antibodies (Bio-Rad) at room temperature for 2 h. High-sensitivity ECL kit (Millipore) was used to visualize the signal of protein bands.

**Table 3:** Antibodies used in this study.

**Antibody**	**Dilution**	**RRIDs**	**Catalogue numbers**	**Source**
GAD1	1:2000	AB_10681171	ab97739	Abcam
EAAT2	1:1000	AB_2813769	ab178401	Abcam
AADC	1:500	AB_304145	ab3905	Abcam
MAOA	1:1000	AB_2137251	10539-1-AP	Proteintech
SERT	1:1000	AB_2813768	ab181034	Abcam
TH	1:200	AB_1310786	ab75875	Abcam
VMAT2	1:1000	AB_1952699	ab70808	Abcam
GLUL	1:500	AB_1127501	sc-74430	Santa Cruz
SNAT1	1:500	AB_2190396	sc-137032	Santa Cruz
β-actin	1:10000	AB_2289225	60008-1-Ig	Proteintech
GAPDH	1:10000	AB_2107436	60004-1-Ig	Proteintech

Abbreviations: RRIDs: research resource identifiers; TH: tyrosine 3-hydroxylase; VMAT2: vesicular monoamine transporter 2.

### Statistical analyses

All results were presented as means ± s.e.m. The statistical analyses of all data were conducted with SPSS 21.0 software. The differences between the two groups were assessed using independent-sample *t*-tests throughout the study. Significant level for all tests was set at P<0.05. All bands were analyzed with Image J. GraphPad Prism 7.0 was used to plot the results of statistical analyses. Key enzymes and transporters were searched via Kyoto Encyclopedia of Genes and Genomes (KEGG, http://www.genome.jp/kegg/).
